# Ethyl 5-methyl-1-(4-nitro­phen­yl)-1*H*-1,2,3-triazole-4-carboxyl­ate

**DOI:** 10.1107/S1600536811033940

**Published:** 2011-08-27

**Authors:** Hoong-Kun Fun, Ching Kheng Quah, Nithinchandra, Balakrishna Kalluraya

**Affiliations:** a X-ray Crystallography Unit, School of Physics, Universiti Sains Malaysia, 11800 USM, Penang, Malaysia; b Department of Studies in Chemistry, Mangalore University, Mangalagangotri, Mangalore 574 199, India

## Abstract

In the title compound, C_12_H_12_N_4_O_4_, the 1,2,3-triazole ring and the nitro group form dihedral angles of 37.93 (5) and 8.97 (12)°, respectively, with the phenyl ring. The mol­ecular structure is stabilized by an intra­molecular C—H⋯O hydrogen bond, which generates an *S*(6) ring motif. In the crystal, mol­ecules are linked by C—H⋯N hydrogen bonds into layers lying parallel to (100). The crystal structure is further consolidated by π–π [centroid–centroid distance = 3.6059 (6) Å] inter­actions.

## Related literature

For general background to and the biological activity of 1,2,3-triazole derivatives, see: Sherement *et al.* (2004 ▶); Danoun *et al.* (1998 ▶); Manfredini *et al.* (2000 ▶); Biagi *et al.* (2004 ▶). For standard bond-length data, see: Allen *et al.* (1987 ▶). For hydrogen-bond motifs, see: Bernstein *et al.* (1995 ▶). For the stability of the temperature controller used for the data collection, see: Cosier & Glazer (1986 ▶). For related structures, see: Fun, Quah, Chandrakantha *et al.* (2011 ▶); Fun, Quah, Nithinchandra *et al.* (2011 ▶).
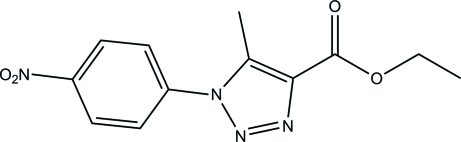


## Experimental

### 

#### Crystal data

C_12_H_12_N_4_O_4_
*M* *_r_* = 276.26
Monoclinic, 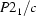

*a* = 13.5309 (3) Å
*b* = 7.3014 (2) Å
*c* = 12.6058 (3) Å
β = 99.574 (1)°
*V* = 1228.04 (5) Å^3^
*Z* = 4
Mo *K*α radiation
μ = 0.12 mm^−1^
*T* = 100 K
0.50 × 0.16 × 0.16 mm

#### Data collection

Bruker SMART APEXII CCD diffractometer
Absorption correction: multi-scan (*SADABS*; Bruker, 2009 ▶) *T*_min_ = 0.944, *T*_max_ = 0.982
16800 measured reflections
4469 independent reflections
3699 reflections with *I* > 2σ(*I*)
*R*_int_ = 0.021

#### Refinement

*R*[*F* ^2^ > 2σ(*F* ^2^)] = 0.040
*wR*(*F* ^2^) = 0.114
*S* = 1.03
4469 reflections
183 parameters
H-atom parameters constrained
Δρ_max_ = 0.41 e Å^−3^
Δρ_min_ = −0.30 e Å^−3^

### 

Data collection: *APEX2* (Bruker, 2009 ▶); cell refinement: *SAINT* (Bruker, 2009 ▶); data reduction: *SAINT*; program(s) used to solve structure: *SHELXTL* (Sheldrick, 2008 ▶); program(s) used to refine structure: *SHELXTL*; molecular graphics: *SHELXTL*; software used to prepare material for publication: *SHELXTL* and *PLATON* (Spek, 2009 ▶).

## Supplementary Material

Crystal structure: contains datablock(s) global, I. DOI: 10.1107/S1600536811033940/hb6375sup1.cif

Structure factors: contains datablock(s) I. DOI: 10.1107/S1600536811033940/hb6375Isup2.hkl

Supplementary material file. DOI: 10.1107/S1600536811033940/hb6375Isup3.cml

Additional supplementary materials:  crystallographic information; 3D view; checkCIF report

## Figures and Tables

**Table 1:** Hydrogen-bond geometry (Å, °)

*D*—H⋯*A*	*D*—H	H⋯*A*	*D*⋯*A*	*D*—H⋯*A*
C1—H1*A*⋯N3^i^	0.95	2.59	3.5243 (12)	168
C5—H5*A*⋯N2^ii^	0.95	2.60	3.2347 (12)	125
C5—H5*A*⋯N3^ii^	0.95	2.54	3.4127 (12)	154
C10—H10*B*⋯O4	0.98	2.48	3.0936 (12)	120

Symmetry codes: (i) 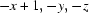; (ii) 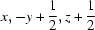.

**Fig. 1.**
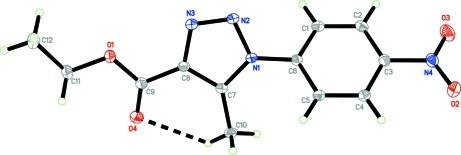
The molecular structure of the title compound showing 50% probability displacement ellipsoids for non-H atoms. The intramolecular hydrogen bond is shown as a dashed line.

**Fig. 2.**
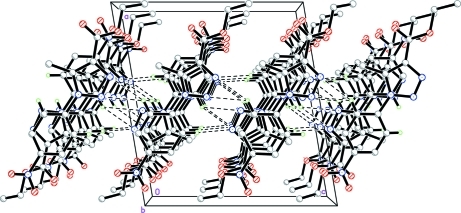
The crystal structure of the title compound, viewed along the b axis. H atoms not involved in hydrogen bonds (dashed lines) have been omitted for clarity.

## supplementary crystallographic information

### Comment

1,2,3-Triazole and its derivatives had attracted considerable attention for the past few decades due to their chemotherapeutical value. Many 1,2,3-triazoles are found to be potent antimicrobial (Sherement *et al.*, 2004) and antiviral agents. Some of them have exhibited antiproliferative and anticancer activities (Danoun *et al.*, 1998). Some 1,2,3-triazoles are used as DNA cleaving agents (Manfredini *et al.*, 2000) and potassium channel activators (Biagi *et al.*, 2004). Prompted by the chemotherapeutic importance of 1,2,3-triazoles and its derivatives, we synthesized the title compound.

In the title molecule, Fig. 1, the 1,2,3-triazole ring (N1-N3/C7/C8, maximum deviation of 0.003 (1) Å at atoms N2 and N3) and the nitro group (O2/O3/N4) form dihedral angles of 37.93 (5) and 8.97 (12)°, respectively, with the phenyl ring (C1-C6). Bond lengths (Allen *et al.*, 1987) and angles are within normal ranges and are comparable to related structures (Fun, Quah, Chandrakantha *et al.*, 2011; Fun, Quah, Nithinchandra *et al.*, 2011). The molecular structure is stabilized by an intramolecular C41-H10B···O4 hydrogen bond (Table 1), which generates an *S*(6) ring motif (Fig. 1, Bernstein *et al.*, 1995).

In the crystal structure, Fig. 2, molecules are linked *via* intermolecular C1–H1A···N3, C5–H5A···N2 and C5–H5A···N3 hydrogen bonds (Table 1) into two-dimensional planes parallel to (100). π-π stacking interactions between the centroids of C1-C6 phenyl ring (Cg1) and N1-N3/C7/C8 triazole ring (Cg2), with Cg1···Cg2^iii^ distance of 3.6059 (6) Å [symmetry code: (iii) 1-X,-1/2+Y,1/2-Z] are observed.

### Experimental

1-Azido-4-nitrobenzene (15 g) was treated with ethyl acetoacetate (8.3 g) in methanol (75 ml) and the mixture was cooled to 273 K. Sodium methoxide (3.5 g) was added under inert atmosphere to the above mixture and stirred at ambient temperature for 8 h. Progress of the reaction was monitored by TLC (ethyl acetate/n-hexane, 2:3, v/v). After completion of the reaction, the mixture was poured on to ice cold water. The precipitated solid was filtered, washed with water and recrystallized from methanol. Colourless plates of (I) were obtained from DMF by slow evaporation.

### Refinement

All H atoms were positioned geometrically and refined using a riding model with C–H = 0.95-0.99 Å and *U*_iso_(H) = 1.2 or 1.5 *U*_eq_(C). A rotating-group model was applied for the methyl groups.

### Crystal data

**Table:**

C_12_H_12_N_4_O_4_	*F*(000) = 576
*M**_r_* = 276.26	*D*_x_ = 1.494 Mg m^−^^3^
Monoclinic, *P*2_1_/*c*	Mo *K*α radiation, λ = 0.71073 Å
Hall symbol: -P 2ybc	Cell parameters from 8459 reflections
*a* = 13.5309 (3) Å	θ = 3.1–32.6°
*b* = 7.3014 (2) Å	µ = 0.12 mm^−^^1^
*c* = 12.6058 (3) Å	*T* = 100 K
β = 99.574 (1)°	Plate, colourless
*V* = 1228.04 (5) Å^3^	0.50 × 0.16 × 0.16 mm
*Z* = 4	

### Data collection

**Table:**

Bruker SMART APEXII CCD diffractometer	4469 independent reflections
Radiation source: fine-focus sealed tube	3699 reflections with *I* > 2σ(*I*)
graphite	*R*_int_ = 0.021
φ and ω scans	θ_max_ = 32.6°, θ_min_ = 3.1°
Absorption correction: multi-scan (*SADABS*; Bruker, 2009)	*h* = −20→18
*T*_min_ = 0.944, *T*_max_ = 0.982	*k* = −11→8
16800 measured reflections	*l* = −19→19

### Refinement

**Table:**

Refinement on *F*^2^	Primary atom site location: structure-invariant direct methods
Least-squares matrix: full	Secondary atom site location: difference Fourier map
*R*[*F*^2^ > 2σ(*F*^2^)] = 0.040	Hydrogen site location: inferred from neighbouring sites
*wR*(*F*^2^) = 0.114	H-atom parameters constrained
*S* = 1.03	*w* = 1/[σ^2^(*F*_o_^2^) + (0.0579*P*)^2^ + 0.3698*P*] where *P* = (*F*_o_^2^ + 2*F*_c_^2^)/3
4469 reflections	(Δ/σ)_max_ = 0.001
183 parameters	Δρ_max_ = 0.41 e Å^−^^3^
0 restraints	Δρ_min_ = −0.30 e Å^−^^3^

### Special details

**Table:**

Experimental. The crystal was placed in the cold stream of an Oxford Cryosystems Cobra open-flow nitrogen cryostat (Cosier & Glazer, 1986) operating at 100.0 (1) K.
Geometry. All esds (except the esd in the dihedral angle between two l.s. planes) are estimated using the full covariance matrix. The cell esds are taken into account individually in the estimation of esds in distances, angles and torsion angles; correlations between esds in cell parameters are only used when they are defined by crystal symmetry. An approximate (isotropic) treatment of cell esds is used for estimating esds involving l.s. planes.
Refinement. Refinement of F^2^ against ALL reflections. The weighted R-factor wR and goodness of fit S are based on F^2^, conventional R-factors R are based on F, with F set to zero for negative F^2^. The threshold expression of F^2^ > 2sigma(F^2^) is used only for calculating R-factors(gt) etc. and is not relevant to the choice of reflections for refinement. R-factors based on F^2^ are statistically about twice as large as those based on F, and R- factors based on ALL data will be even larger.

### Fractional atomic coordinates and isotropic or equivalent isotropic displacement parameters (Å^2^)

**Table:**

	*x*	*y*	*z*	*U*_iso_*/*U*_eq_	
O1	0.17538 (5)	0.16665 (10)	−0.05384 (5)	0.01804 (15)	
O2	0.81492 (6)	0.19872 (12)	0.56912 (6)	0.02603 (18)	
O3	0.88912 (6)	0.04601 (13)	0.45702 (7)	0.02874 (19)	
O4	0.14766 (5)	0.02184 (11)	0.09686 (6)	0.02182 (16)	
N1	0.45814 (6)	0.12739 (11)	0.18124 (6)	0.01308 (15)	
N2	0.46803 (6)	0.17529 (12)	0.07841 (6)	0.01609 (16)	
N3	0.37872 (6)	0.17308 (12)	0.02083 (6)	0.01568 (16)	
N4	0.81564 (6)	0.12105 (13)	0.48264 (7)	0.01954 (17)	
C1	0.63468 (7)	0.05446 (13)	0.22772 (7)	0.01570 (17)	
H1A	0.6336	0.0113	0.1564	0.019*	
C2	0.72344 (7)	0.05470 (13)	0.30098 (7)	0.01654 (17)	
H2A	0.7841	0.0126	0.2807	0.020*	
C3	0.72162 (7)	0.11789 (13)	0.40463 (7)	0.01516 (17)	
C4	0.63467 (7)	0.17883 (13)	0.43757 (7)	0.01519 (17)	
H4A	0.6358	0.2197	0.5093	0.018*	
C5	0.54574 (7)	0.17947 (13)	0.36424 (7)	0.01414 (16)	
H5A	0.4852	0.2209	0.3850	0.017*	
C6	0.54695 (6)	0.11829 (13)	0.25977 (7)	0.01296 (16)	
C7	0.36061 (6)	0.09299 (12)	0.18864 (7)	0.01266 (16)	
C8	0.31097 (7)	0.12254 (13)	0.08482 (7)	0.01365 (16)	
C9	0.20306 (7)	0.09756 (13)	0.04465 (7)	0.01549 (17)	
C10	0.32346 (7)	0.03491 (14)	0.28808 (7)	0.01660 (18)	
H10A	0.3749	−0.0389	0.3327	0.025*	
H10B	0.2624	−0.0381	0.2686	0.025*	
H10C	0.3087	0.1434	0.3284	0.025*	
C11	0.06813 (7)	0.14784 (16)	−0.09678 (8)	0.0212 (2)	
H11A	0.0276	0.1941	−0.0440	0.025*	
H11B	0.0510	0.0175	−0.1116	0.025*	
C12	0.04675 (8)	0.25753 (17)	−0.19890 (8)	0.0252 (2)	
H12A	−0.0243	0.2459	−0.2301	0.038*	
H12B	0.0879	0.2116	−0.2502	0.038*	
H12C	0.0627	0.3866	−0.1830	0.038*	

### Atomic displacement parameters (Å^2^)

**Table:**

	*U*^11^	*U*^22^	*U*^33^	*U*^12^	*U*^13^	*U*^23^
O1	0.0132 (3)	0.0239 (4)	0.0165 (3)	−0.0003 (3)	0.0008 (2)	0.0035 (3)
O2	0.0228 (4)	0.0363 (5)	0.0175 (3)	−0.0044 (3)	−0.0011 (3)	0.0009 (3)
O3	0.0157 (3)	0.0370 (5)	0.0330 (4)	0.0053 (3)	0.0024 (3)	0.0051 (4)
O4	0.0167 (3)	0.0304 (4)	0.0189 (3)	−0.0049 (3)	0.0043 (2)	0.0031 (3)
N1	0.0132 (3)	0.0159 (4)	0.0106 (3)	−0.0003 (3)	0.0035 (2)	0.0001 (3)
N2	0.0153 (3)	0.0219 (4)	0.0116 (3)	−0.0003 (3)	0.0039 (3)	0.0019 (3)
N3	0.0146 (3)	0.0198 (4)	0.0130 (3)	−0.0003 (3)	0.0036 (3)	0.0011 (3)
N4	0.0155 (4)	0.0227 (4)	0.0197 (4)	−0.0016 (3)	0.0009 (3)	0.0060 (3)
C1	0.0166 (4)	0.0165 (4)	0.0150 (4)	0.0002 (3)	0.0056 (3)	−0.0009 (3)
C2	0.0143 (4)	0.0169 (4)	0.0194 (4)	0.0013 (3)	0.0057 (3)	0.0007 (3)
C3	0.0136 (4)	0.0156 (4)	0.0159 (4)	−0.0009 (3)	0.0013 (3)	0.0030 (3)
C4	0.0159 (4)	0.0164 (4)	0.0133 (3)	−0.0003 (3)	0.0027 (3)	0.0012 (3)
C5	0.0141 (4)	0.0158 (4)	0.0130 (3)	0.0006 (3)	0.0037 (3)	0.0001 (3)
C6	0.0130 (4)	0.0132 (4)	0.0128 (3)	−0.0004 (3)	0.0028 (3)	0.0008 (3)
C7	0.0136 (4)	0.0121 (4)	0.0129 (3)	−0.0009 (3)	0.0041 (3)	−0.0005 (3)
C8	0.0141 (4)	0.0152 (4)	0.0124 (3)	−0.0004 (3)	0.0042 (3)	−0.0003 (3)
C9	0.0149 (4)	0.0175 (4)	0.0142 (4)	0.0006 (3)	0.0027 (3)	−0.0014 (3)
C10	0.0176 (4)	0.0198 (4)	0.0134 (4)	−0.0026 (3)	0.0057 (3)	0.0010 (3)
C11	0.0130 (4)	0.0282 (5)	0.0213 (4)	−0.0002 (4)	−0.0007 (3)	0.0024 (4)
C12	0.0207 (5)	0.0312 (6)	0.0220 (4)	0.0025 (4)	−0.0015 (4)	0.0047 (4)

### Geometric parameters (Å, °)

**Table:**

O1—C9	1.3348 (11)	C4—C5	1.3901 (12)
O1—C11	1.4685 (11)	C4—H4A	0.9500
O2—N4	1.2303 (12)	C5—C6	1.3933 (12)
O3—N4	1.2246 (12)	C5—H5A	0.9500
O4—C9	1.2103 (12)	C7—C8	1.3849 (12)
N1—C7	1.3616 (11)	C7—C10	1.4878 (12)
N1—N2	1.3705 (10)	C8—C9	1.4751 (13)
N1—C6	1.4258 (11)	C10—H10A	0.9800
N2—N3	1.3022 (11)	C10—H10B	0.9800
N3—C8	1.3686 (11)	C10—H10C	0.9800
N4—C3	1.4730 (12)	C11—C12	1.5029 (14)
C1—C2	1.3879 (13)	C11—H11A	0.9900
C1—C6	1.3962 (13)	C11—H11B	0.9900
C1—H1A	0.9500	C12—H12A	0.9800
C2—C3	1.3897 (13)	C12—H12B	0.9800
C2—H2A	0.9500	C12—H12C	0.9800
C3—C4	1.3846 (13)		
			
C9—O1—C11	114.50 (8)	N1—C7—C8	103.28 (7)
C7—N1—N2	111.11 (7)	N1—C7—C10	125.27 (8)
C7—N1—C6	131.17 (7)	C8—C7—C10	131.44 (8)
N2—N1—C6	117.71 (7)	N3—C8—C7	109.38 (8)
N3—N2—N1	107.24 (7)	N3—C8—C9	123.56 (8)
N2—N3—C8	108.98 (7)	C7—C8—C9	127.01 (8)
O3—N4—O2	124.49 (9)	O4—C9—O1	125.10 (9)
O3—N4—C3	117.77 (9)	O4—C9—C8	122.48 (8)
O2—N4—C3	117.74 (8)	O1—C9—C8	112.41 (8)
C2—C1—C6	119.36 (8)	C7—C10—H10A	109.5
C2—C1—H1A	120.3	C7—C10—H10B	109.5
C6—C1—H1A	120.3	H10A—C10—H10B	109.5
C1—C2—C3	118.52 (8)	C7—C10—H10C	109.5
C1—C2—H2A	120.7	H10A—C10—H10C	109.5
C3—C2—H2A	120.7	H10B—C10—H10C	109.5
C4—C3—C2	122.51 (8)	O1—C11—C12	107.67 (8)
C4—C3—N4	118.52 (8)	O1—C11—H11A	110.2
C2—C3—N4	118.97 (8)	C12—C11—H11A	110.2
C3—C4—C5	119.11 (8)	O1—C11—H11B	110.2
C3—C4—H4A	120.4	C12—C11—H11B	110.2
C5—C4—H4A	120.4	H11A—C11—H11B	108.5
C4—C5—C6	118.85 (8)	C11—C12—H12A	109.5
C4—C5—H5A	120.6	C11—C12—H12B	109.5
C6—C5—H5A	120.6	H12A—C12—H12B	109.5
C5—C6—C1	121.66 (8)	C11—C12—H12C	109.5
C5—C6—N1	120.02 (8)	H12A—C12—H12C	109.5
C1—C6—N1	118.27 (8)	H12B—C12—H12C	109.5
			
C7—N1—N2—N3	0.42 (10)	C7—N1—C6—C1	142.62 (10)
C6—N1—N2—N3	179.36 (8)	N2—N1—C6—C1	−36.07 (12)
N1—N2—N3—C8	−0.58 (10)	N2—N1—C7—C8	−0.07 (10)
C6—C1—C2—C3	0.41 (14)	C6—N1—C7—C8	−178.83 (9)
C1—C2—C3—C4	0.58 (14)	N2—N1—C7—C10	179.02 (9)
C1—C2—C3—N4	−179.20 (8)	C6—N1—C7—C10	0.27 (15)
O3—N4—C3—C4	171.06 (9)	N2—N3—C8—C7	0.56 (11)
O2—N4—C3—C4	−8.86 (13)	N2—N3—C8—C9	−177.07 (9)
O3—N4—C3—C2	−9.16 (13)	N1—C7—C8—N3	−0.28 (10)
O2—N4—C3—C2	170.93 (9)	C10—C7—C8—N3	−179.30 (9)
C2—C3—C4—C5	−0.85 (14)	N1—C7—C8—C9	177.24 (9)
N4—C3—C4—C5	178.93 (8)	C10—C7—C8—C9	−1.78 (17)
C3—C4—C5—C6	0.12 (14)	C11—O1—C9—O4	1.16 (14)
C4—C5—C6—C1	0.87 (14)	C11—O1—C9—C8	−179.05 (8)
C4—C5—C6—N1	−176.36 (8)	N3—C8—C9—O4	166.28 (9)
C2—C1—C6—C5	−1.14 (14)	C7—C8—C9—O4	−10.92 (16)
C2—C1—C6—N1	176.14 (8)	N3—C8—C9—O1	−13.51 (13)
C7—N1—C6—C5	−40.05 (14)	C7—C8—C9—O1	169.29 (9)
N2—N1—C6—C5	141.26 (9)	C9—O1—C11—C12	171.11 (9)

### Hydrogen-bond geometry (Å, °)

**Table:**

*D*—H···*A*	*D*—H	H···*A*	*D*···*A*	*D*—H···*A*
C1—H1A···N3^i^	0.95	2.59	3.5243 (12)	168
C5—H5A···N2^ii^	0.95	2.60	3.2347 (12)	125
C5—H5A···N3^ii^	0.95	2.54	3.4127 (12)	154
C10—H10B···O4	0.98	2.48	3.0936 (12)	120

Symmetry codes: (i) −*x*+1, −*y*, −*z*; (ii) *x*, −*y*+1/2, *z*+1/2.
